# A pre-operative predictive score to evaluate the feasibility of complete cytoreductive surgery in patients with epithelial ovarian cancer

**DOI:** 10.1371/journal.pone.0187245

**Published:** 2017-11-08

**Authors:** Marion Chesnais, Fabrice Lecuru, Myriam Mimouni, Charlotte Ngo, Arnaud Fauconnier, Cyrille Huchon

**Affiliations:** 1 EA 7285 Clinical Risks and Safety on Women's Health, University Versailles-Saint-Quentin en Yvelines, Montigny-le-Bretonneux, France; 2 Gynecologic Oncology Centre Paris Descartes- Hôpital Européen Georges Pompidou, APHP, Paris, France; 3 Faculté de Médecine, Université Paris Descartes, Sorbonne Paris Cité, Paris, France; 4 INSERM UMR S 1124, Faculté de Médecine, Université Paris Descartes, Paris, France; 5 Department of Gynecology and Obstetrics, CHI Poissy-Saint-Germain, Poissy, France; The University of Texas MD Anderson Cancer Center, UNITED STATES

## Abstract

**Objective:**

Postoperative residual tumor is the major prognostic factor in ovarian cancer. The feasibility of complete cytoreductive surgery is assessed by laparoscopy. Our goal was to develop a predictive score prior to laparoscopy to evaluate the feasibility of complete cytoreductive surgery in patients with epithelial ovarian cancer.

**Methods:**

We developed a score to predict incomplete cytoreductive surgery by performing multiple logistic regressions after bootstrap procedures on data from a retrospective cohort of 247 patients with advanced ovarian cancer. This score was validated on a different population of 45 patients with ovarian cancer.

**Results:**

Four criteria were independently associated with incomplete cytoreduction, confirmed by surgery: BMI ≥ 30 kg/m^2^ (adjusted odds ratio [aOR], 3.07; 95% confidence interval [95% CI], 1.0–9.6), CA125 > 100 IU/L (aOR, 3.99; 95% CI, 1.6–10.1), diaphragmatic and/or omental carcinomatosis by CT-Scan (aOR, 5.82; 95% CI, 2.6–13.1), and positive parenchymal metastases by PET/CT (aOR, 3.59; 95% CI, 1.0–12.8). The 100-point score was based on these criteria. The area-under-the-curve of the score was 0.79 (95% CI, 0.73–0.86). In the validation group, no patient ranked in the high-risk group of incomplete cytoreductive surgery had a complete upfront cytoreductive surgery (95% CI 0–16). Three of 29 patients for whom primary complete cytoreduction was not possible were classified in the group at low risk of incomplete cytoreductive surgery (12%; 95% CI 4–27).

**Conclusion:**

This pre-operative score may be useful for distinguishing which patients may have complete cytoreductive surgery from those who will receive neoadjuvant chemotherapy, while avoiding unnecessary laparoscopy.

## Introduction

Residual tumor size is the main prognostic factor in patients with advanced epithelial ovarian cancer. The aim of cytoreductive surgery is complete cytoreduction, defined as the absence of macroscopic tumor upon completion of the surgery (CC-0, Completeness Cytoreductive score, [1]), as it produces the best outcomes in terms of overall and progression free survival [2, 3, 4, 5].

It is essential in the management of ovarian cancer to identify the patients who will benefit from primary complete cytoreductive surgery and those for whom primary complete resection is impossible (patients then referred for neoadjuvant chemotherapy).

Laparoscopy; with the Fagotti and Fagotti-modified scores; is currently the most common procedure to assess tumor spread and resectability [6, 7, 8, 9]. Laparoscopic evaluation, nevertheless, has limits and may, in some cases, underestimate the extent of the disease [10]. Complete primary cytoreductive surgery will not be achieved for these patients, first classified “resectable” by laparoscopy (Laparoscopy false negative for detecting unresectability). They are then exposed to surgical complications without the benefit of the oncological prognosis; and a delay in initiating chemotherapy.

The objective of our study was to establish a new preoperative predictive score (upstream of laparoscopy), based on clinical, biological, and radiological elements, to evaluate the resectability of peritoneal carcinomatosis in the upfront management of ovarian malignant epithelial tumors.

## Materials and methods

### Study design and patients

We conducted a bicenter retrospective study in consecutive patients managed for epithelial ovarian cancer at two university teaching hospitals. Patients from the Oncological Surgery Center of the Georges Pompidou Hospital (Paris, France) were included between December 1, 2008 and May 30, 2013 and those from the Department of Gynecology and Obstetrics of the Poissy-Saint-Germain Hospital (Poissy, France) between December 1, 2009 and May 30, 2015. Our score was developed using data from the population of the Georges Pompidou Hospital and validated on data from patients of the Poissy-Saint-Germain Hospital. Our study was approved by the Comité d'Ethique de la Recherche en Gynécologie Obstétrique (CEROG n°2016-GYN-1002).

We included all patients who had epithelial cancer of the ovary, tube, or peritoneum. We only used the data on the initial assessment of the disease and primary cytoreductive surgery. We excluded patients with non-epithelial or borderline tumors, and those who did not have initial exploratory or diagnostic surgery. Our database consisted of clinical, biological, and radiological information.

### Gold standard

The diagnostic reference used to confirm the resectability of peritoneal carcinomatosis was median laparotomy for complete cytoreductive surgery and median laparotomy or laparoscopy for unresectability.

Patients were defined as "resectable" when primary cytoreductive surgery was complete (CC0) by median laparotomy. "Unresectable" patients were defined as those who did not receive primary complete cytoreductive surgery including:

- Patients who underwent surgery, but whose final residual tumor was not CC0 (CC1 and more), due to the extent of peritoneal carcinomatosis or comorbidities of the patient.
- Patients who first underwent laparoscopy and were assessed as unresectable, and then referred for neoadjuvant chemotherapy.

Cytoreductive surgery was deemed contraindicated in patients with any of the following laparoscopy criteria: massive involvement of the hepatic pedicle, bowel involvement requiring extensive small-bowel resection, the need for more than two gastrointestinal resections and/or mesenteric resection, suprarenal lymphadenopathy, and massive involvement of the retroperitoneum [11].

### Statistical analysis

We compared patients defined as "resectable" to those defined as "unresectable." For between-group comparisons of qualitative variables, we used the chi-square test or, when the expected sample size was too small, Fisher’s exact test. The univariate analysis of quantitative variables was performed using Student’s t test. Variables significantly associated with incomplete cytoreduction were dichotomized on either side of the best cutoffs identified by receiver-operating characteristic (ROC) curves. Regarding the "age" variable, the cutoff was selected to ensure face validity: ages of 50 and higher seemed clinically relevant to us.

Variables yielding p values < 0.20 by univariate analysis were entered into a multivariate model using backward stepwise logistic regression. The combination of variables exhibiting the strongest independent association with resectability at the p level of < 0.05 was identified.

Bootstrap resampling was performed to assess the robustness of the multivariate model, using 1,000 replications [12]. This resampling procedure allows the identification and elimination of any unstable variables. Robust 95% confidence intervals were then obtained by computing the mean of the results from the iterations. Once a stable logistic regression model was obtained, the C-statistic with its 95% CI was computed.

The ability of the model to classify patients in the correct group was assessed using the c-statistic of the ROC curve (Receiver Operating Curve) and its 95% confidence interval (95% CI). The calibration of the model was assessed using the Hosmer—Lemeshow test.

The stable logistic regression model was used to build a score based on the rounded values of the β coefficients with a multiplicative factor to produce a simple scale. The C-statistics of the rounded score and logistic regression model were compared to verify that the score derived from the model was not statistically different from the logistic regression model. The probability of incomplete cytoreduction associated with each value of the score was calculated and the sensitivity and specificity of various score cut-offs computed.

The score was then applied to the validation population for external validation. Sensitivities, specificities, positive and negative likelihood ratios, and their 95% CI were calculated for the same cut-offs as those selected from the validation population.

We also tested the score in the advanced-stage subgroup (FIGO stages IIIC and IV) in the derivation and validation population.

Stata software version 13.0 (Stata Corp., College Station, TX, USA) was used for the statistical analysis.

## Results

### Patients

**Fig 1:**
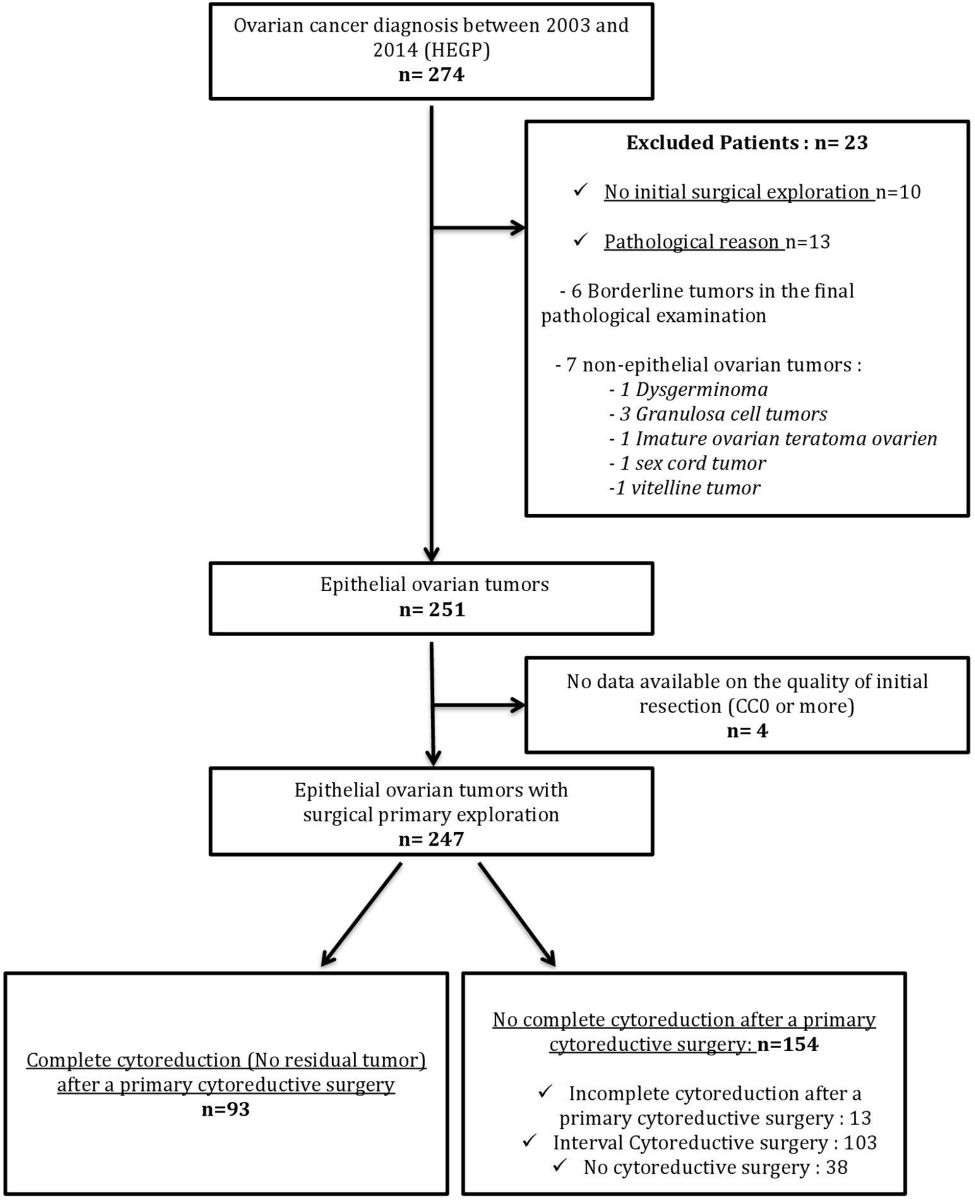
Flow chart for the derivation population. HEGP, Hôpital Européen Georges Pompidou.

During the study period, 274 patients were suspected of having ovarian cancer in the derivation group. Among them, 27 were excluded (10 did not undergo exploratory surgery, six tumors were borderline, seven had non-epithelial tumors, and surgical data were not available for four patients). Of the 247 patients left for the study, 93 (37.7%) had primary complete cytoreductive surgery (CC-0) and 154 (62.3%) were diagnosed as "unresectable" (Fig 1). Table 1 presents the main patient characteristics.

**Table 1:** Patient characteristics in derivation and validation populations.

*Variable*	*Derivation population n = 247*	*Validation population n = 49*	*p value*
**Age** (years) mean +/- SD	62.5 +/- 12.8	59.3 +/- 13.5	0.12
**BMI** (kg/m2) mean +/- SD (212/ 45)	23.8+/- 4.7	25.4 +/- 6.3	0.06
**Gestity** mean +/- SD (176/47)	1.8 +/-1.7	1.8 +/-1.5	0.88
**Parity** mean +/- SD (209/47)	1.6 +/-1.4	1.6 +/-1.4	0.84
**Menopause** n/N (%)	157/188 (83.5)	34/49 (69.4)	0.04
**Smoking** n/N (%)	21 /157 (13.4)	7/47 (14.9)	0.99
Personal history of gynecological cancer* n/N (%)	22/247 (8.9)	4 /49 (8.2)	1
Familial history of gynecological cancer* n/N (%)	63/247 (25.5)	10 /49 (20.4)	0.57
**Initial Ca125 level** (IU/L) mean +/- SD (224/45)	1203+/-1877	1474+/- 3974	0.48
**Albuminemia** mean +/- SD (131/14)	28.2 +/-8.5	33.9+/- 7.6	0.02
**FIGO stage**			0.02
I	32 /244 (13.1)	13 (26.5)	
II	16 /244(6.6)	2 (4.1)	
III	120/244 (49.2)	27 (55.1)	
IV	76 /244 (31.1)	7 (14.3)	
**Histology**			0.6
Serous	136/202 (67.3)	21/28 (75)	
Mucinous	8/202 (3.9)	3/28 (10.7)	
Endometrioid	24 /202 (11.9)	2/28 (7.1)	
Clear cell	10/202 (4.9)	0	
Mixed	3 /202 (1.5)	0	
Undifferentiated	14 /202 (6.9)	1 /28 (3.6)	
Others	7/202 (3.4)	1/28 (3.6)	
**Type of surgical exploration** n/N (%)			0.05
Laproscopy	173/247(70)	35/49(71.4)	
Laparotomy conversion	57/247 (23.1)	6 /49 (12.2)	
Initial laparotomy	17/247 (6.9)	8 /49 (16.3)	
**Size of the original mass** (cm) mean +/- SD (179/37)	9.1 +/- 5.1	12.8 +/- 6.8	<0.01
**Peritoneal carcinomatosis at diagnosis** n/N (%)	162 /240 (67.5)	35 /49 (71.4)	0.71
**Distant metastasis** n/N (%)	60/218 (27.5)	6 /48(12.5)	0.03
**Pleural effusion** n/N (%)	41/218 (18.8)	1/46 (2.2)	<0.01
**Ascites initial volume** (cc) mean +/- SD (181/29)	1014+/- 1558	1142+/- 1605	0.69

Gynecological cancer: Uterus, breast or ovarian cancer; SD, Standard deviation.

The validation sample consisted of 45 patients of Poissy Saint Germain Hospital.

### Univariate and multivariate analyses

The findings from the univariate analysis are reported in Table 2. The proportion of patients with primary complete cytoreductive surgery was not significantly different between the derivation group (93/247, 37.7%, 95% CI, 33–44) and the validation group (20/49, 40.8%, 95% CI, 28–55).

**Table 2:** Univariate analysis of the derivation population.

*Variable*	*Resectable Patients n = 93*	*Unresectable patients n = 154*	*Total population n = 247*	*OR (CI95%)*	*p value*	*c index*
**Clinical Data, n (%)**						
Age> = 50 years	33 (35.5%)	80 (52%)	113 (45.7%)	2.3 (1.17–4.59)	**0.01**	0.56
Obesity (BMI> = 30)	7 (8.5%)	21 (8.2%)	28 (11.3%)	2.06 (0.83–5.14)	0.11	0.54
Menopause	52 (76.5%)	107 (87.5%)	159 (64.4%)	2.15 (0.98–4.74)	0.05	0.56
Personnal history of gynecological cancer	14 (15%)	8 (5.2%)	22 (8.9%)	0.31 (0.12–0.78)	**0.01**	0.45
Familial history of gynecological cancer	30 (32.3%)	33 (21.4%)	63 (22.5%)	0,57 (0.32–1.03)	0.06	0.45
**Biological Data**						
* **Albuminemia*						
mean+/SD	27.1+/- 10.3	28.8 +/-7.2				
< = 20g/L (n, %)	15 (30%)	9 (11%)	24 (9.7%)	0.29 (0.11–0.75)	**0.01**	0.41
* **CA125*						
mean +/-SD	914 +/-198	1314 +/-161				
> = 100 UI/L (n, %)	43 (50.6%)	124 (89.2%)	167 (67.6%)	8.07 (3.78–17.26)	**<0.01**	0.70
**Radiological Data n (%)**						
* **Ultrasound/Scan*						
Ascites	41 (46.1%)	109 (79.6%)	150 (60.7%)	4.56 (2.43–8.56)	**<0.01**	0.67
Omentum and/or diaphragmatic carcinomatosis	23 (27.1%)	97 (77.6%)	120 (48.6%)	9.3 (4.5–19.5)	**<0.01**	0.75
- Omentum	17 (20.2%)	83 (68.6%)	100 (40.5%)	8.61 (4.07–18.19)	**<0.01**	0.74
- Diaphragmatic	13 (16.5%)	65 (55.1%)	78 (31.6%)	6.23 (2.92–13.28)	**<0.01**	0.69
Abdominal/ pelvic nodes	18 (21.4%)	55 (41%)	73 (29.6%)	2.55 (1.35–4.84)	**0.01**	0.6
Digestive	10 (12.5%)	42 (31.6%)	52 (21.1%)	3.37 (1.55–7.32)	**<0.01**	0.6
- Colon	6 (7.3%)	23 (18%)	29 (11.7%)	2.77 (1.06–7.24)	**0.03**	0.55
- Small intestine	3 (3.7%)	12 (9.4%)	15 (6.1%)	2.72 (0.74–10.0)	0.12	0.53
- Mesentery	3 (3.7%)	15 (11.7%)	18 (7.3%)	3.47 (0.96–12.56)	**0.04**	0.54
Metastasis	10 (12.8%)	50 (35.7%)	60 (24.3%)	6.35 (2.98–13.51)	**<0.01**	0.69
Pleural effusion	4 (5.1%)	37 (26.4%)	41 (16.6%)	6.65 (2.18–20.27)	**<0.01**	0.61
* **Positive PET /CT*						
Mass	45 (90%)	78 (83%)	123 (49.8%)	0.54 (0.41–0.52)	0.26	0.46
Carcinomatosis	30 (37%)	86 (77.4%)	116 (47.0%)	5.81 (0.18–1.59)	**<0.01**	0.7
Diaphragmatic	11 (20.8%)	43 (48.9%)	54 (21.9%)	6.23 (2.87–11.74)	**<0.01**	0.64
Lymphadenopathy	12 (15%)	36 (29.5%)	48 (19.4%)	2.37 (1.61–8.28)	**0.02**	0.57
Metastasis	8 (10.4%)	45 (35.4%)	53 (21.5%)	4.73 (2.02–11.10)	**<0.01**	0.69

BMI, Body Mass Index; Gynecological cancer: Uterus, breast or ovarian cancer; SD, Standard deviation; PET/CT, Positron emission tomography–computed tomography; OR, Odds Ratio; 95% CI, 95% confidence interval.

The multivariate analysis identified four variables independently associated with incomplete cytoreduction, namely, BMI ≥ 30 kg/m^2^ (adjusted odds ratio [aOR], 3.07; 95% confidence interval [95% CI], 1.0–9.6), CA125 > 100 IU/L (aOR, 3.99; 95% CI, 1.6–10.1), diaphragmatic and/or omental carcinomatosis by CT-Scan (aOR, 5.82; 95% CI, 2.6–13.1), and positive parenchymal metastases by PET/CT (aOR, 3.59; 95% CI, 1.0–12.8). The C-statistic was 0.82 (95% CI 0.76-.88). The bootstrap procedure established that all model variables were robust. The calibration of the predictive model was satisfactory, with a non-significant Hosmer—Lemeshow test (p = 0.51).

### Cytoreduction feasibility score

The score was built from the variables identified by multivariate logistic regression (Table 3). The C-statistic of the score was 0.79 (95% CI, 0.73–0.86).

**Table 3:** Score and risk groups.

**Variable**	**Points**
BMI≥ 30 kg/m^2^	20
Ca125≥ 100 IU/L	25
Diaphragmatic and/or omental carcinomatosis by CT-Scan	35
Positive parenchymal metastases by PET/CT	20
	Score = sum of points/100
**Risk group**	**Predicted risk of incomplete cytoreduction (95% IC), %**
Low < 25	18.9 (9.5–34.2)
Middle 25–6	64.6 (54.6–73.4)
High > 60	86.4 (73.3–93.6)

BMI, Body Mass Index; PET/CT, Positron emission tomography–computed tomography; 95% CI, 95% confitdence interval.

We next used the score to predict incomplete cytoreduction. The group at low risk for incomplete cytoreduction was composed of the patients whose score was < 25. These patients accounted for 20.9% of all patients. The probability of incomplete cytoreduction in the low-risk group was 18.9% (95% CI 9.5–34.2). The group at high risk for incomplete cytoreduction was composed of the patients whose score was > 60, i.e., 24.9% of all patients. The probability of incomplete cytoreduction in the high-risk group was 86.4% (95% CI, 73.3–93.6). With a cutoff of < 25, the sensitivity for predicting incomplete cytoreduction was 93.5% (95% CI, 87.1–96.8) and the negative likelihood ratio 0.15 (95% CI, 0.07–0.33). With a cutoff of > 60, the specificity for predicting incomplete cytoreduction was 91.4% (95% CI, 82.5–96) and the positive likehood ratio 4.1 (95% IC, 1.9–9.3).

In the advanced-stage subgroup (FIGO stages IIC and IV, n = 167), the probability of incomplete cytoreduction in the high-risk group was 89.5% (77/86; 95% CI, 81.3–94.4). Half of the patients classified as part of the low-risk group of incomplete cytoreduction had a primary complete cytoreductive surgery (3/6; 95% CI, 18.8–81.2).

We next tested the score on the validation group and the results were consistent with those of the derivation group. No patient ranked in the high-risk group of incomplete cytoreductive surgery had a primary complete surgery (97.5% CI 0–16). Among patients classified by the score in the low-risk group of incomplete cytoreduction, 12% did not have primary complete cytoreduction (3/29; 95% CI 4–27). Concerning the advanced-stage subgroup of the validation population (n = 33), no patient in the high-risk group of incomplete cytoreduction had a primary complete surgery (0/9; 97.5% CI, 0–30). 25% of patients classified in the low-risk group of incomplete cytoreduction had a primary complete surgery (1/4; 95% CI, 4.6–70.0).

## Discussion

**Fig 2:**
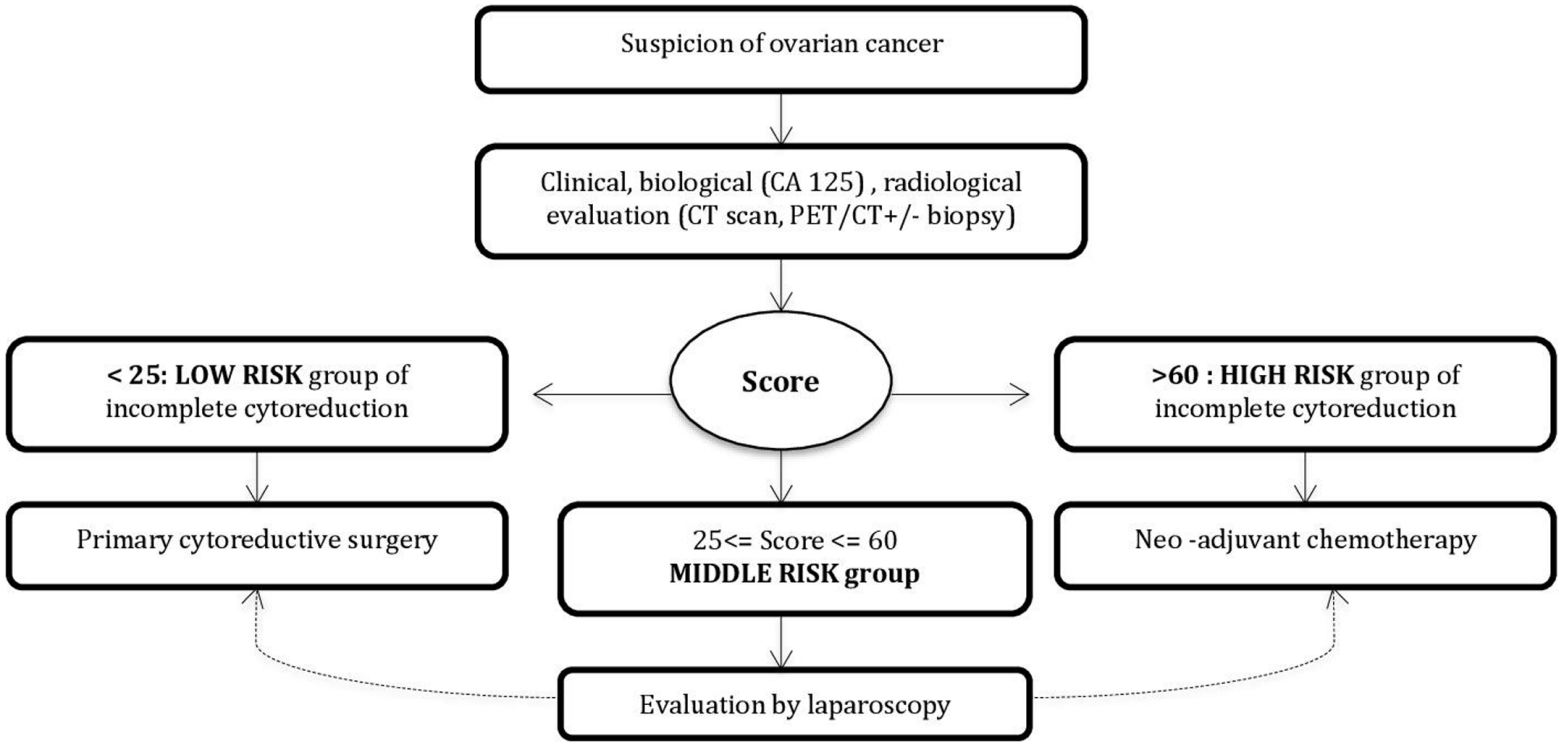
Decision algorithm.

We developed a 100 point-score to predict the resectability of peritoneal carcinomatosis in patients with ovarian cancer. This score is based on four criteria: a clinical criterion, BMI ≥ 30 kg/m^2^ (aOR, 3.07; 95% CI, 1.0–9.6), a biological criterion, CA125 >100 IU/L (aOR, 3.99; 95% CI, 1.6–10.1), and two radiological criteria: diaphragmatic and/or omental carcinomatosis by CT-Scan (aOR, 5.82; 95% CI, 2.6–13.1), and positive parenchymal metastases by PET/CT (aOR, 3.59; 95% CI, 1.0–12.8). Patients whose score was > 60 were at high risk for incomplete cytoreduction (86.4%, 95% CI, 73.3–93.6). Patients with scores < 25 were at low risk for incomplete cytoreduction (18.9%, 95%CI 9.5–34.2). For patients with a score between 25 and 60, the risk of incomplete cytoreduction was intermediate (64.6%, 95% CI 54.6–73.4). We propose a new surgical decision algorithm based on the score (Fig 2).

In their 2006 pilot study, Fagotti *et al*. compared their laparoscopic score to laparotomy in 64 patients with stage III or IV ovarian cancer. The overall accuracy rate of laparoscopy was 90%, the negative predictive value 100%, and the positive predictive value 87% [6]. Recently a review of the literature has been done comparing existing criteria to predict the outcome of cytoreductive surgery. Laparoscopy seems to have a highly valuable role, since it is safe, reproducible, and provides organ specific tumor sampling to investigate new therapeutics [8]. Moreover, it reduces the number of futile laparotomies by four [9]. That is why laparoscopic evaluation, using the Fagotti and Fagotti modified scores, is currently the standard procedure in the diagnostic work-up in some institutes. However, it is only performed when there is doubt about the resectability of tumor metastases in others.

Laparoscopy evaluation has several limitations: it is directly related to the surgeon’s experience and there is a risk of intra-peritoneal tumor rupture and trocar metastases during surgery. In addition, anatomical and technical limitations make the exploration difficult in some areas (diaphragmatic dorsal area, gastrosplenic ligament, lymph nodes and mesenteric retractation) [8]. Finally, general anesthesia may induce complications. These limitations explain the misclassifications that occur when using laparoscopy to predict resectability, sometimes resulting in an underestimation. In a Cochrane review of 2014, covering seven articles, between 36 and 73% of patients were deemed resectable during exploratory laparoscopy. However, the rate of false negatives (patients for whom optimal cytoreduction was not possible) ranged from 4 to 31% [10].

Chereau *et al*. compared various methods to assess resectability in 61 patients with epithelial ovarian cancer, including the PCI, Fagotti modified, and Eisenkop scores, the surgical complexity score of Aletti, and FIGO classification. In the subgroup of FIGO stages III and IV, the best discrimination was found using the Fagotti modified score (AUC = 0.69) [13]. However, most of these scores were designed to predict optimal cytoreductive surgery, defined as residual tumor of less than 1 cm in diameter, whereas the current standard is complete cytoreductive surgery, defined as the absence of any visible tumor.

Several groups have proposed composite scoring systems comprised of clinical, biological, and radiological elements. Most were designed using a smaller population than ours, which consisted of 247 patients [14, 15, 16]. In 2011, Gerestein *et al*. developed a score based on platelet levels and scanner data of a population with a complete cytoreduction rate comparable to ours (42.5%). The AUC of this score was 0.67 [16].

More recently, Suidan *et al*. and Dessapt *et al*. developed composite scores with similar overall predictive accuracy (c-statistic of 0.76 and 0.78 respectively), but without external validation [17, 18]. Our study was carried out in two different institutions, one for the derivation of the score and the other one for its external validation, demonstrating its reproducibility.

Limits of our study are explained by several biases. First, collection bias may have occurred, given the retrospective design. Our score was built on many variables, which exposes us to a risk of type 1 error. There were high rates of missing data for several of these variables. However, it was mostly "Missing Completely at Random" data, which induce a loss of precision and power in the analysis, but not true bias [19]. Classification bias could have also occurred in our study. Indeed, the surgeon's experience is a key element in ovarian cancer management: for the same patient, one surgeon may achieve complete resection whereas a less experienced colleague will not succeed [13]. Our score included diaphragmatic and omental carcinomatosis in CT scan. Usually these findings are not hinder for complete cytoreduction. But when they are present, they often reflect a greater extent of carcinomatosis. That is probably why this variable is significantly associated with incomplete cytoreduction in the univariate and multivariate analyses.

Furthermore, we decided to include all FIGO stages, unlike most studies that consider only advanced ones, because higher stage or more extensive disease cannot be excluded before the surgical exploration. Moreover, by applying our algorithm to the advanced-stage subgroup, we obtained consistent results: respectively 89.5% and 100% of patients in the high-risk group of incomplete cytoreduction in the derivation and in the validation population did not have a complete surgery.

In our study, when patients were considered "unresectable" by laparoscopy, they received neoadjuvant chemotherapy. Among these cases, there is a risk of false positives: resectable patients evaluated as unresectable by laparoscopy, addressed to neoadjuvant chemotherapy instead of surgery. In order to reduce these misclassifications, we used reliable and robust criteria to assess unresectability [11]. In laparoscopy, moreover, false-positives are rare. In two Fagotti studies in which laparotomy was performed in all patients, the negative predictive value of laparoscopy was 100% [20, 21].

Finally, the definition of risk groups does not allow classification of more than half of the patients (57.7% in the intermediate risk group). For these patients, a laparoscopic evaluation needs to be performed. However, projecting onto 100 patients, our score would classify 42 patients to be of low or high risk of incomplete cytoreduction, and therefore 42 patients would avoid a laparoscopic evaluation.

## Conclusion

We have developed a 100-point score which is reproducible, original, and would be easy to use in routine practice. Patients would be classified into one of the three risk groups of incomplete cytoreduction following clinical, biological, and radiological evaluation. Patients classified as high risk would immediately be referred for neoadjuvant chemotherapy. Low- risk patients may benefit from primary complete cytoreductive surgery. No laparoscopy would therefore be necessary for these two risk groups. Intermediate-risk patients would still benefit from laparoscopy to assess the resectability of the disease.

## Supporting information

S1 Table Database. (ZIP)
